# Decellularized extracellular matrix restores Fibronectin/Integrin β1 balance through extracellular vesicles to rejuvenate chondrocytes and alleviate osteoarthritis progression

**DOI:** 10.1016/j.jot.2025.10.011

**Published:** 2025-12-20

**Authors:** Aoyuan Fan, Zhiying Pang, Zheng Liu, Feng Yin, Yiming Wang

**Affiliations:** a Department of Joint Surgery, Shanghai East Hospital, Tongji University School of Medicine, Shanghai, 200120, China; b Shanghai Institute of Stem Cell Research and Clinical Translation, Shanghai, 200025, China

**Keywords:** Decellularized extracellular matrix, Extracellular vesicles, Osteoarthritis, Cellular senescence, Fibronectin, Integrin β1

## Abstract

**Introduction:**

Osteoarthritis (OA) is a prevalent degenerative joint disease driven largely by chondrocyte senescence. Extracellular vesicle (EV)-based therapies have emerged as a promising strategy; however, the extensive stem-cell expansion required to obtain therapeutic EV doses unavoidably erodes their potency.

**Objectives:**

Leveraging our prior finding that decellularized extracellular matrix (dECM) rejuvenates stem cells during in vitro expansion, we further investigate whether dECM could resolve the current bottleneck in EV therapy by preserving therapeutic efficacy even in late-passage cells.

**Methods:**

Human adipose-derived stromal cells (hADSCs) were expanded to passage 15 on either tissue culture plastic (TCP) or dECM, and their EVs were isolated. We first interrogated the capacity of dECM-primed EVs to counteract chondrocyte senescence and ER stress in vitro, then validated their therapeutic impact in a rat OA model. Mechanistic insight was pursued through proteomic profiling, followed by loss- and gain-of-function studies using pharmacologic inhibitors and targeted knockdown.

**Results:**

Late-passage EVs generated under dECM (dECM-P15-EVs) surpassed those under TCP (TCP-P15-EVs) in alleviating chondrocyte senescence and ER stress. In vivo, dECM-P15-EVs attenuated cartilage degradation more effectively than their conventionally cultured counterparts. Proteomics revealed dECM-P15-EVs were enriched in both FN and its receptor integrin β1 (ITGB1). Either pharmacologic blockade or siRNA-mediated knockdown of FN in dECM or of ITGB1 in EV-producing cells abrogated the anti-senescence and chondro-protective benefits of dECM-P15-EVs. Further experiments implicated FN/ITGB1 transfer as a critical step in re-activating downstream SIRT1 signaling.

**Conclusion:**

By reinstating FN/ITGB1 homeostasis and reinvigorating SIRT1-dependent pathways, dECM-P15-EVs effectively counteract chondrocyte senescence and OA progression—offering a scalable, senescence-resistant platform for next-generation EV therapy.

The Translational Potential of this Article: Producing the large quantities of EVs required for clinical OA therapy necessitates prolonged expansion of stem cells, which inevitably blunts EV efficacy. dECM culture restores the potency of EVs without additional biosafety concerns. Thus, dECM-P15-EVs offer strong translational promise as an advanced, EV-centric OA therapy that overcomes current limitations.

**The translational potential of this article:**

Producing the large quantities of EVs required for clinical OA therapy necessitates prolonged expansion of stem cells, which inevitably blunts EV efficacy. dECM culture restores the potency of EVs without additional biosafety concerns. Thus, dECM-P15-EVs offer strong translational promise as an advanced, EV-centric OA therapy that overcomes current limitations.

## Graphical abstract

**Figure:**
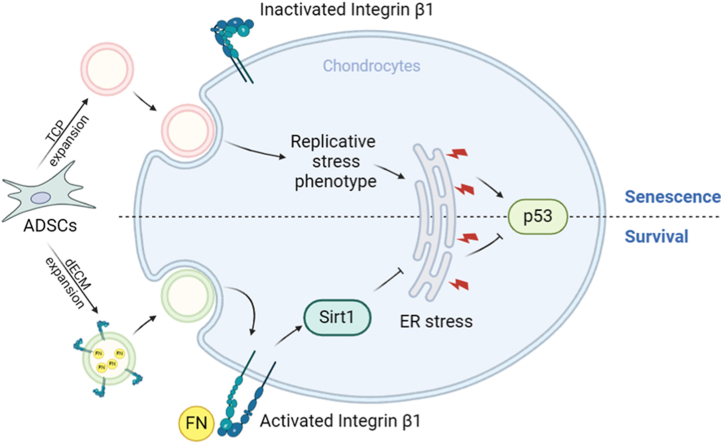

## 1 Introduction

As the most prevalent joint disease [1], osteoarthritis (OA) is one of the leading causes of disability in the elderly, affecting over half of the individuals aged 65 and older [2]. Cellular senescence, a fundamental hallmark of aging, has been linked to OA pathogenesis [3]. The predominant pathological feature of OA is progressive articular cartilage destruction, characterized by chondrocyte senescence and concomitant extracellular matrix (ECM) degradation [3]. The reparative efforts induce the proliferation of hypertrophic chondrocytes and matrix protein synthesis. However, aberrant protein synthesis leads to endoplasmic reticulum (ER) stress with the consequent cartilage matrix loss [4]. Therefore, targeting ER stress to inhibit chondrocyte senescence constitutes an important clinical avenue for OA therapy.

Current conservative treatments provide symptomatic relief but fail to halt or reverse structural joint deterioration. Mesenchymal stromal cell (MSC)-based injectable therapies have shown encouraging disease-modifying potential, with extracellular vesicles (EVs) emerging as a particularly attractive cell-free alternative [5]. Compared with their parent MSCs, EVs exhibit lower immunogenicity, negligible tumorigenic risk, and versatile regenerative properties [6]. Meta-analyses have documented pleiotropic EV-mediated benefits in OA, including anti-inflammatory, anti-apoptotic, and anti-catabolic effects [7,8]; however, only sparse data address the direct modulation of OA-associated senescence by EVs [9].

Clinically relevant EV yields require extensive MSC expansion to high passage [10,11], which inevitably erodes their therapeutic potency [9]. Long-term passaged MSCs yield EVs with altered protein and miRNA profiles that lack the rejuvenating potency of EVs secreted by early-passage MSCs [[12], [13], [14], [15], [16]]. Thus, preserving the functional integrity of MSCs—and consequently their EVs—is paramount for optimizing OA therapy.

In vivo, MSCs reside within a three-dimensional niche that provides biochemical and biophysical cues essential for self-renewal and function [17]. Recapitulating this niche ex vivo can sustain proliferative capacity and preserve therapeutic competence [18,19]. Decellularized extracellular matrix (dECM), formed by removing the cellular components from native cells or tissues, constitutes a biomimetic scaffold rich in structural proteins (collagen, fibronectin [FN], elastin) and instructive signals [20,21]. Our previous studies have demonstrated that dECM culture could effectively rejuvenate MSCs [[22], [23], [24]]; in particular, the major dECM constituent FN markedly influences the behavior of expanded cells [24]. Yet, whether FN can preserve the therapeutic potency of MSC-derived EVs against passage-induced decline remains to be elucidated.

Integrin β1 (ITGB1) is one of the principal and classic receptors for FN, thereby orchestrating cell adhesion, mechano-transduction, and survival signaling [25]. Genetic abrogation of the FN-α5β1 integrin interaction in articular cartilage aggravates osteoarthritis in mice [26]. Via FN/integrin-mediated interactions, dECM transmits environmental cues that modulate cell fate [27]; yet how the FN–ITGB1 axis governs chondrocyte senescence and its downstream mechanisms remain unclear.

Sirtuin 1 (SIRT1), an NAD^+^-dependent deacetylase, is a well-characterized anti-senescence factor that modulates metabolism through deacetylation of histone and non-histone substrates [28]. SIRT1 signaling is strongly involved in the regulation of OA progression [29,30]. Especially under the influence of ER stress, SIRT1 restrains excessive glycolysis and suppresses the ensuing inflammatory cascade driven by metabolic reprogramming [28]. Moreover, SIRT1 participates in integrin recycling and FN-integrin-mediated signaling [[31], [32], [33], [34]]. We therefore postulate that FN/ITGB1 and SIRT1 may act synergistically to counteract chondrocyte senescence.

In this study, late-passage human adipose-derived MSCs (hADSCs, P15) were cultured on hADSC-derived dECM or conventional tissue culture plastic (TCP), and their EVs were isolated and compared. Functional assays revealed that dECM-P15-EVs exerted superior therapeutic efficacy in attenuating OA progression. Proteomic profiling identified FN and ITGB1 as enriched cargoes in dECM-P15-EVs. Mechanistically, delivery of FN and ITGB1 via dECM-P15-EVs activated SIRT1 signaling, thereby mitigating chondrocyte senescence and cartilage degeneration. These findings establish dECM culture as a practical strategy to generate potent, senescence-resistant EV therapeutics for OA.

## 2 Material and methods

### 2.1 Ethics statement

This study was carried out in compliance with the Helsinki Declaration and approved by the Institutional Review Board of Shanghai East Hospital; written informed consent was obtained from all patients prior to human sample collection. All animal procedures were approved by the Tongji University School of Medicine Animal Care and Use Committee. The ethical approval number for this project issued by our institution is TJBB03921401 and DFSC-2019(CR)-03.

### 2.2 Cell culture

Human articular chondrocytes (Sciencell, Catalog #4650) were cultured in Dulbecco's Modified Eagle Medium (HyClone, SH30022, USA) with 10 % FBS (Tianhang, 141215, China), 100 U/ml penicillin and 100 μg/ml streptomycin.

For hADSCs isolation, subcutaneous adipose tissue from 10 volunteers (5 males, 5 females; 34 ± 4 years, 28–40 years) was minced and digested with 0.1 % collagenase II (Sigma–Aldrich, C6885, USA) for 2 h followed by 0.1 % trypsin (Gibco, 25200056, USA) for 0.5 h at 37 °C. Cells were cultured in iCell Primary MSC Culture System (iCell, PriMed-iCell-012, China).

Lentiviral siRNA was used to generate FN-knockdown hADSCs and ITGB1-knockdown hADSCs according to our previous study [24]. Briefly, hADSCs were transduced at a suitable multiplicity of infection with scramble sequence-containing vector or siRNA vectors using Lipofectamine™ 3000 kit (Thermofisher, L3000001, USA). For FN, ITGB1 and SIRT1 inhibition, FN inhibitor sc-202156 (100 μM, 24 h), ITGB1 inhibitor ATN-161 (20 μM, 2 h) and SIRT1 inhibitor EX-527(10 μM, 2 h) were used for pre-conditioning of chondrocytes before further treatment.

### 2.3 β-gal staining

For cell senescence evaluation, β-gal staining was conducted according to the manufacturer's instruction (Beyotime, C0602, China). Briefly, cells were fixed for 15 min at room temperature. After rinsing, cells were stained with β-gal staining solution overnight at 37 °C. Images were acquired using Inverted Fluorescence Microscope (Olympus, IX51, Japan).

### 2.4 Immunofluorescent (IF) staining

For IF staining, cells were fixed and blocked before antibody incubation. Cells were then incubated with primary antibodies against p53 (PTG, 60283-2-Ig, mouse, 1:200, China), GPR78 (PTG, 11587-1-AP, rabbit, 1:200, China), CHOP (PTG,15204-1-AP, rabbit, 1:200, China), PTGS2 (PTG, 12375-1-AP, rabbit, 1:200, China), integrin α3 (ITGA3; Abcam, Ab131055, rabbit, 1:200, USA), ITGB1 (Abcam, Ab24693, mouse, 1:200, USA; PTG, 12594-1-AP, rabbit, 1:400, China), FN (Abcam, Ab268020, rabbit, 1:300, USA) or SIRT1 (Abcam, Ab189494, rabbit, 1: 300, USA) followed by incubation with Cy3-labeled goat anti-mouse secondary antibody (Aspen, AS-1111, 1:100, China), CoraLite488-conjugated goat anti-rabbit (PTG, SA00013-2, 1:200, China), Cy3-labeled Goat anti-Rabbit secondary antibody (Aspen, AS-1109, 1:100, China) or CoraLite488-conjugated goat anti-mouse (PTG, SA00013-1, 1:200, China). Cells were stained with DAPI (Sigma, D8417, USA) for 5 min. Images were acquired using Inverted Fluorescence Microscope (Olympus, IX51, Japan).

For IF evaluation, ImageJ was used to calculate the positive area of both certain protein and DAPI using uniformed thresholds in each group. The ratio was used for further evaluation.

### 2.5 Western blot analysis

For western blot analysis, total protein was extracted from cell lysis using RIPA buffer (Aspen, AS1004, China) and after quantified with BCA protein assay kit (Aspen, AS1086, China), total protein from each sample was separated using SDS-PAGE gels. Bands were transferred onto PVDF Membrane (Millipore, IPVH00010, USA). After blocking, the membrane was incubated with primary monoclonal antibodies targeting p53 (Abcam, Ab183544, rabbit, 1:500, USA), FN (Abcam, Ab45688, rabbit, 1:1000, USA), ITGA3 (Affbiotech, AF5182, rabbit, 1:500, China)), ITGB1 (Abclonal, A19072, rabbit, 1:500, USA), SIRT1 (CST, #9475, rabbit, 1:1000, USA) or GAPDH (Abcam, Ab181602, rabbit, 1:10000, USA) in 5 % BSA in TBST buffer at 4 °C overnight, followed by the secondary antibody of HRP-conjugated goat anti-rabbit (Aspen, AS-1107, 1: 10000, China)) for 1 h. ECL reagents (Aspen, AS-1059, China)) were used for exposure.

### 2.6 dECM preparation and ADSCs culture

dECM was prepared following a previous protocol described in our previous study [24]. For the TCP-EV culture, standard tissue-culture polystyrene plates (TCPs) were pre-coated with 0.2 % gelatin (Millipore Sigma, 1288485, Germany) for 1h, following 1 % glutaraldehyde (Millipore Sigma, 340855, Germany) and 1M ethanolamine (Millipore Sigma, 15014, Germany) for 30 min prior to hADSCs seeding. For the dECM-EV culture, when hADSCs seeded on the pre-coated TCPs reached 90 % confluence, 250 μM L-ascorbic acid phosphate (Millipore Sigma, 49752, Germany) was added to the culture medium for seven days. 0.5 % Triton X-100 (Thermo Scientific, USA, 28313) containing 20 mM ammonium hydroxide (Sargent-Welch, 470300-204, USA) was added for 5 min. After cell removal, dECM was stored at 4 °C in phosphate buffered solution (PBS) containing 100 U/mL penicillin, 100 μg/mL streptomycin, and 0.25 μg/mL fungizone until use.

### 2.7 EVs isolation and characterization

After harvesting, the hADSC medium was spun at 2000 g for 30 min to remove cells and debris, passed through a 0.22 μm filter, mixed with Total exosome isolation reagent (Invitrogen, 4478359, USA), and centrifuged at 10 000 g for 10 min; the pellet was resuspended in PBS. Donor EVs were pooled before use. TEM assessed morphology, NTA measured size, and western blot probed CD9 (Abcam, Ab92726, 1:1000, USA), CD63 (Abcam, Ab216130, 1:1000, USA) and HSP70 (Abcam, Ab181606, 1:2000, USA). For uptake assays, EVs were labeled with PKH26 (Sigma–Aldrich, PKH26GL, USA) for 5 min, quenched with 1 % BSA, and incubated with chondrocytes for 12 h. Phalloidin-FITC (Yeasen, 40735ES75, 1:100, China) and DAPI (5 min) were applied without secondary antibody. Images were acquired using Inverted Fluorescence Microscope (Olympus, IX51, Japan).

### 2.8 qPCR

For qPCR experiment, total RNA was extracted using TRIpure Total RNA Extraction Reagent (ELK Biotechnology, EP013, USA) and was reverse-transcribed using EntiLink™ 1st Strand cDNA Synthesis Kit (ELK Biotechnology, EQ003, USA). qPCR reaction was conducted using EnTurbo™ SYBR Green PCR SuperMix (ELK Biotechnology, EQ001, USA). qPCR amplification was performed using a StepOne™ Real-Time PCR System (Life technologies, USA) according to the manufacturer's instructions. The PCR primers used include GAPDH: 5′- CGT CTT CAC CAC CAT GGA GA-3′ and 5′- CGG CCA TCA CGC CAC AGT TT-3’; ACAN: 5′- CAG GAG AGA GCC ATC AGA GG-3′ and 5′- CTG AAA GAG GGA CCC TTG G-3’; COL2A1: 5′- CGT CTT CAC CAC CAT GGA GA-3′ and 5′- CGG CCA TCA CGC CAC AGT TT-3’; SOX9: 5′- CGG AGG AAG TCG GTG AAG-3′ and 5′- GGG AGA TGT GCG TCT GCT-3’.

### 2.9 Reactive oxygen species (ROS) analysis

ROS level was measured by flow cytometry using a ROS analysis kit (Beyotime, S0033, China) based on the manufacturer's instructions. Briefly, cells were detached and incubated with 0.5 μL DCFH-DA solution for 20 min at 37 °C. After rinse and centrifuge, fluorescence was measured by a FACS AriaII (BD Biosciences, USA) using the FCS Express software package (De Novo Software, USA).

### 2.10 Animals

For the destabilization of the medial meniscus (DMM) operation, rats were anesthetized with 1 % pentobarbital (35–40 mg/kg) and given ketoprofen (5 mg/kg s.c.). After shaving and sterilizing the knee, a 4 mm incision was made from the patellar pole to the tibial plateau. The capsule was opened medial to the patellar tendon, and the fat pad dissected. In DMM rats the medial meniscotibial ligament was transected and the sham rats had no further intervention.

All rats were randomly divided into 5 groups: (1) Control group (sham rats receiving saline injection into the knee joint); (2) OA group (DMM rats receiving saline injection into the knee joint); (3) OA + TCP-P5-EVs group (DMM rats receiving the injection of 50 μL 1 × 10¹⁰ particles/mL TCP-P5-EVs in PBS into the knee joint); (4) OA + TCP-P15-EVs group (DMM rats receiving the injection of 50 μL 1 × 10¹⁰ particles/mL TCP-P15-EVs into the knee joint); (5) OA + dECM-P15-EVs group (DMM rats receiving the injection of 50 μL 1 × 10¹⁰ particles/mL dECM-P15-EVs into the knee joint). Injections were given twice weekly for 4 weeks starting 4 weeks post-surgery; knees were harvested at 8 weeks.

### 2.11 Histology, immunohistochemical (IHC) and IF staining

Dissected rat knee joints were fixed in 4 % paraformaldehyde (Sinopharm, 80096618, China) overnight at 4 °C and decalcified in 10 % EDTA (Sinopharm, 10009617, China). Paraffin-embedded knee joints were sectioned at 5 μm thickness, and stained with hematoxylin, Safranin O solution Fast Green solution (Solarbio, G1371, China). Grading was performed by calculating OARSI scores [35]. Images were acquired using Inverted Fluorescence Microscope (Olympus, IX51, Japan).

For IHC staining, sections were deparaffinated, rehydrated, and blocked before antibody staining. Sections were then incubated with primary antibodies against Collagen II (PTG, 28459-1-AP, 1: 300, China) followed by the horseradish peroxidase (HRP)-conjugated Goat anti Rabbit secondary antibody (Aspen, AS-1107, 1: 200, China).

For IF staining, sections were then incubated with primary antibodies against p53 (PTG, 10442-1-AP, rabbit, 1:200, China), FN (Abcam, Ab268020, rabbit, 1: 300, USA) and ITGB1 (PTG, 12594-1-AP, rabbit, 1: 400, China), followed by incubation with a secondary antibody conjugated to Cy3-labeled Goat anti-Rabbit secondary antibody (Servicebio, GB21303, 1:100, China). Nuclei were counterstained with DAPI, and images were acquired using Inverted Fluorescence Microscope (Olympus, IX51, Japan).

### 2.12 Proteomics

Proteomic analysis was performed using Tandem Mass Tags. Total protein from TCP-P15-EVs and dECM-P15-EVs was extracted; aliquots were taken for concentration determination and SDS-PAGE, while the remainder underwent trypsin digestion and labeling. Equal amounts of labeled samples were pooled, separated by chromatography, and analyzed by LC-MS/MS. After quality assessment and preprocessing, quantitative data were subjected to expression and functional analyses. Functional annotation employed multiple databases; differential proteins were analyzed for GO terms, pathways, and interactions. Correlation, heat-map clustering, and Venn analyses were conducted across comparison groups.

### 2.13 Flow cytometry

For the EVs uptake and ITGB1 transfer study, EVs were incubated with PKH26 (Sigma–Aldrich, PKH26GL, USA) for 5 min and 1 % BSA was used to stop the reaction. PKH26 labeled EVs were resuspended in basal medium and incubated with chondrocytes for 12 h. EVs uptake and ITGB1 level was measured by flow cytometry using a FITC anti-rat ITGB1 Antibody (Invitrogen, 11-0291-82, USA) based on the manufacturer's instructions. Briefly, cells were detached and incubated with FITC anti-rat ITGB1 Antibody for 20 min at 4 °C. After rinse and centrifuge, fluorescence was measured by a CytoFLEX (Beckman, USA).

### 2.14 Statistical analysis

Data were presented as mean ± standard deviation (SD). The statistical significance of differences between the two groups was assessed using two-tailed Student's t-tests. The statistical significance of differences among more than two groups was assessed using one-way ANOVAs with Tukey's multiple comparison tests, or two-way ANOVAs with Sidak's multiple comparison tests. All data represent mean ± SD. *P* values less than 0.05 were considered significant (∗*P* < 0.05, ∗∗*P* < 0.01, ∗∗∗*P* < 0.001). Statistical analysis was conducted by GraphPad Prism.

## 3 Results

### 3.1 ER stress inhibition mitigates chondrocyte senescence

**Fig. 1:**
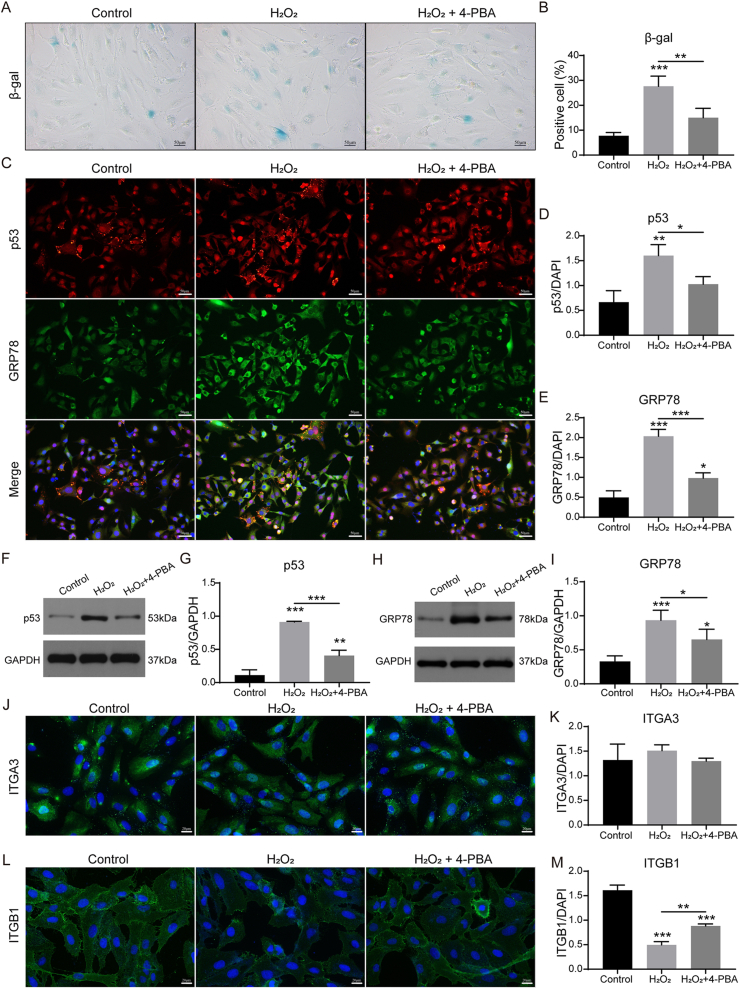
ER stress inhibition mitigates chondrocyte senescence. Chondrocytes were treated for 24 h with 100 mM H_2_O_2_ ± 200 ng/ml 4-PBA. (A) β-gal staining of chondrocytes and (B) quantification (scale bar, 50 μm; n = 3); (C) Immunostaining of p53 and GRP78 in chondrocytes and (C,D) quantifications (DAPI labels nuclei; scale bar, 50 μm; n = 3); (F, H) Western blot of p53 and GRP78 protein level in chondrocytes and (G, I) quantifications (GAPDH served as loading control; n = 3); (J,L) Immunostaining of ITGA3 and ITGB1 in chondrocytes and (K,M) quantifications (DAPI labels nuclei; scale bar, 20 μm; n = 3). The statistical significance was assessed using one-way ANOVAs with Tukey's multiple comparison tests (∗*P* < 0.05, ∗∗*P* < 0.01, ∗∗∗*P* < 0.001).

Persistent ER stress is a recognized driver of senescence [4]. Serial passaging or H_2_O_2_ (100 mM, 24 h) increased β-gal-positive cell percentage and senescence marker p53 expression, while simultaneously elevating the ER-stress marker GRP78 expression (Fig. 1A–I; Supplementary Fig. 1). Pharmacologic suppression of ER stress with 4-phenylbutyric acid (4-PBA) reduced GRP78 and, consequently, β-gal-positive cell percentage and p53 expression (Fig. 1A–I; Supplementary Fig. 1). These results established ER stress as an upstream, reversible trigger of chondrocyte senescence.

Meanwhile, previous study has implicated ITGB1 in alleviating ER stress and apoptosis in high-glucose induced myocardial cells [36]. We therefore examined the expression of ITGB1 in H_2_O_2_-challenged chondrocytes. H_2_O_2_ selectively down-regulated ITGB1 without significantly altering ITGA3, and 4-PBA restored ITGB1 expression (Fig. J–M). These data position ITGB1 as an ER-stress-responsive regulator whose recovery is necessary for the anti-senescence effect.

### 3.2 dECM culture preserves EV identity

**Fig. 2:**
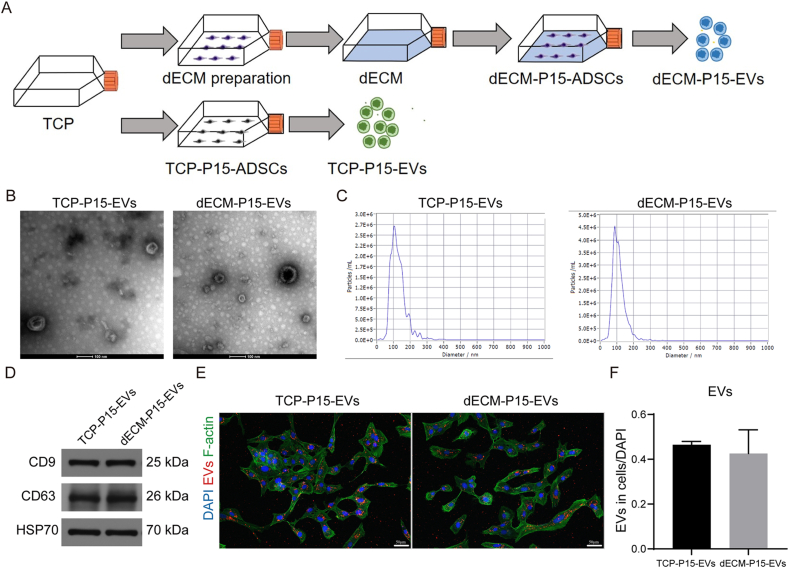
dECM culture preserves EV identity. TCP-P15-EVs and dECM-P15-EVs were acquired. (A) Schematic of both EV preparation; (B) TEM analysis of both EVs; (C) NTA analysis of both EVs; (D) Western blot for surface marker CD9, CD63, and HSP70 in both EVs; (E) EVs uptake in chondrocytes treated with both EVs and (F) quantification of intracellular EVs (DAPI labels nuclei; scale bar, 50 μm; n = 3). The statistical significance was assessed using two-tailed Student's unpaired t-tests. Data represent mean ± SD.

Experimental scheme of TCP-P15-EVs and dECM-P15-EVs preparation was illustrated (Fig. 2A). TEM revealed cup-shaped vesicles for both TCP-P15-EVs and dECM-P15-EVs (Fig. 2B); NTA recorded mean diameters of 127.2 nm and 110.6 nm, respectively (Fig. 2C). The presence of exosomal markers CD9, CD63, and HSP70 (Fig. 2D) and absence of GM130 and Calnexin (Supplementary Fig. 2D) were confirmed by Western blot analysis. Uptake assays showed equivalent chondrocyte internalization (Fig. 2E and F). Thus, dECM culture yields EVs without altering their basic EV characterization including morphology, size, marker profile, or uptake.

### 3.3 dECM-P15-EVs curb chondrocyte senescence and cartilage loss

Accumulating data show EVs curb OA progression [6], yet large-scale production demands extensive passaging [37] and may influence the potency of EVs [9,12,13]. We previously reported that dECM preserves MSC rejuvenation [19,[22], [23], [24]]. Consistent with this, dECM-cultured hADSCs displayed lower CHOP and GRP78 levels than TCP controls (Supplementary Fig. 2A–C), indicating reduced ER stress.

**Fig. 3:**
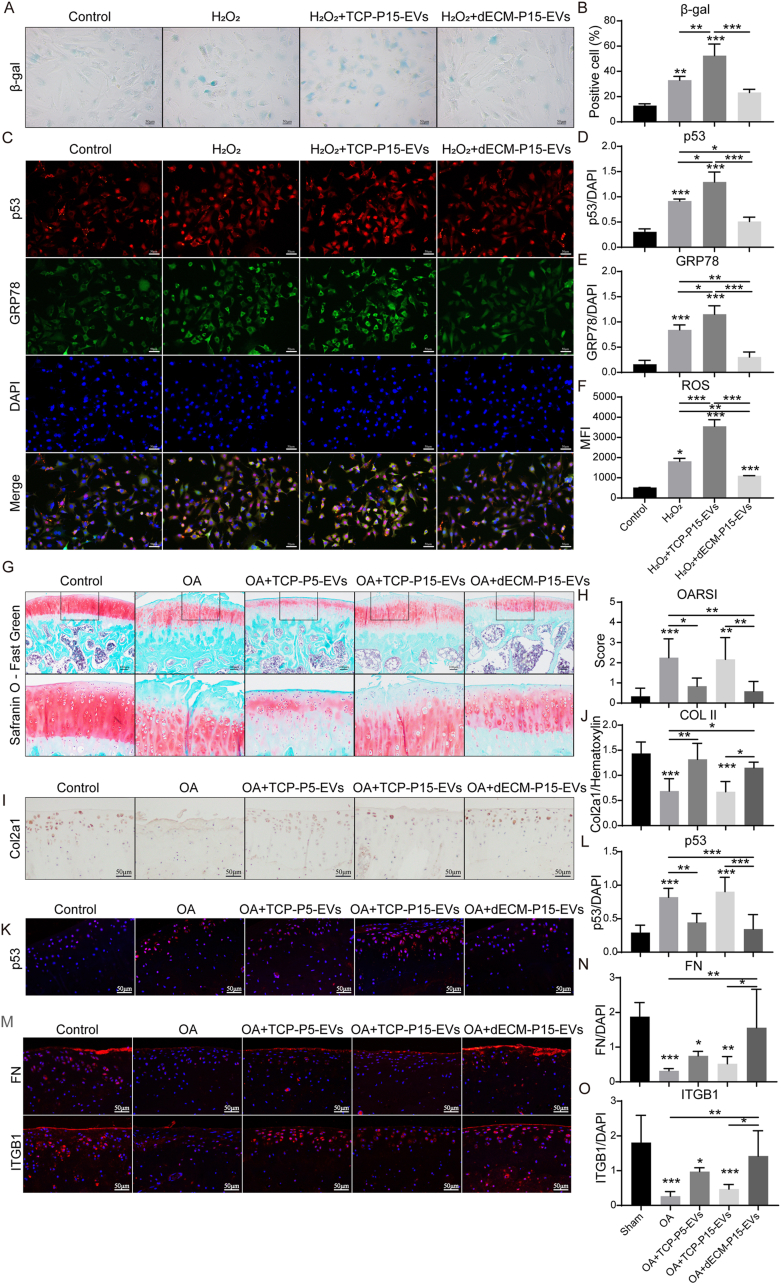
dECM-P15-EVs curb chondrocyte senescence and cartilage loss. Chondrocytes were treated with 100 mM H_2_O_2_ ± 10 μg/ml TCP-P5-EVs, TCP-P15-EVs or dECM-P15-EVs for 24 h. (A) β-gal staining of chondrocytes and (B) quantification (scale bar, 50 μm; n = 3); (C) Immunostaining of p53 and GRP78 in chondrocytes and (D,E) quantifications (DAPI labels nuclei; scale bar, 20 μm; n = 3); (F) ROS level of chondrocytes; TCP-P5-EVs, TCP-P15-EVs, or dECM-P15-EVs (50 μL, 1 × 10¹⁰ particles/mL) were injected into the knee joint of DMM rats (G) Safranin O/Fast Green staining of the cartilage samples and (H) quantification of cartilage erosion (scale bar, 100 μm; n = 6); (I) Immunohistochemical staining of Col2a1 in cartilage samples and (J) quantification (scale bar, 50 μm; n = 6); (K) Immunostaining of p53 in cartilage samples and (L) quantification (scale bar, 50 μm; n = 6); (M) Immunostaining of FN and ITGB1 in cartilage samples and (N,O) quantifications (scale bar, 50 μm; n = 6). The statistical significance was assessed using one-way ANOVAs with Tukey's multiple comparison tests (∗*P* < 0.05, ∗∗*P* < 0.01, ∗∗∗*P* < 0.001).

To investigate whether hADSCs cultured on the dECM can alleviate chondrocyte senescence via EV delivery, we compared P15-EVs from TCP and dECM in H_2_O_2_-stressed chondrocyte senescence model. TCP-P15-EVs raised β-gal positive cell percentage and p53 expression, whereas dECM-P15-EVs restored both levels to baseline (Fig. 3A–D) and lowered GRP78 expression and ROS level (Fig. 3C–E,F), implicating reduced ER and oxidative stress. Moreover, dECM-P15-EVs matched the anti-senescence potency of TCP-P5-EVs from early-passage hADSCs, suppressing H_2_O_2_-induced senescence and restoring ACAN, COL2A1, and SOX9 expression (Supplementary Fig. 3A–G). In the OA model, intra-articular injection of TCP-P5-EVs or dECM-P15-EVs attenuated cartilage damage as demonstrated in Safranin O Fast Green staining (OARSI score, Fig. 3G and H), whereas TCP-P15-EVs were ineffective. Immunostaining revealed higher Col2a1 and lower p53 in cartilage treated with dECM-P15-EVs than with TCP-P15-EVs (Fig. 3I–L).

In a rabbit osteochondral defect model, MSC-EV treatment restored ITGB1 expression in the regenerated cartilage tissue [38]. We therefore examined FN and ITGB1 in cartilage treated with dECM-P15-EVs and found robust restoration of both proteins (Fig. 3M−O), suggesting their effective delivery and functional uptake by chondrocytes could be the possible mechanism for the anti-senescence effect of dECM-P15-EVs (Fig. 3M−O).

Collectively, dECM could rejuvenate hADSC-derived EVs, enabling them to suppress the ER stress-driven chondrocyte senescence and promote cartilage degeneration. To further elucidate the cargo difference of dECM-P15 EVs compared to TCP-P15-EVs, we further conducted proteomic analysis.

### 3.4 Proteomic profiling of EVs from TCP- vs dECM-cultured hADSCs

**Fig. 4:**
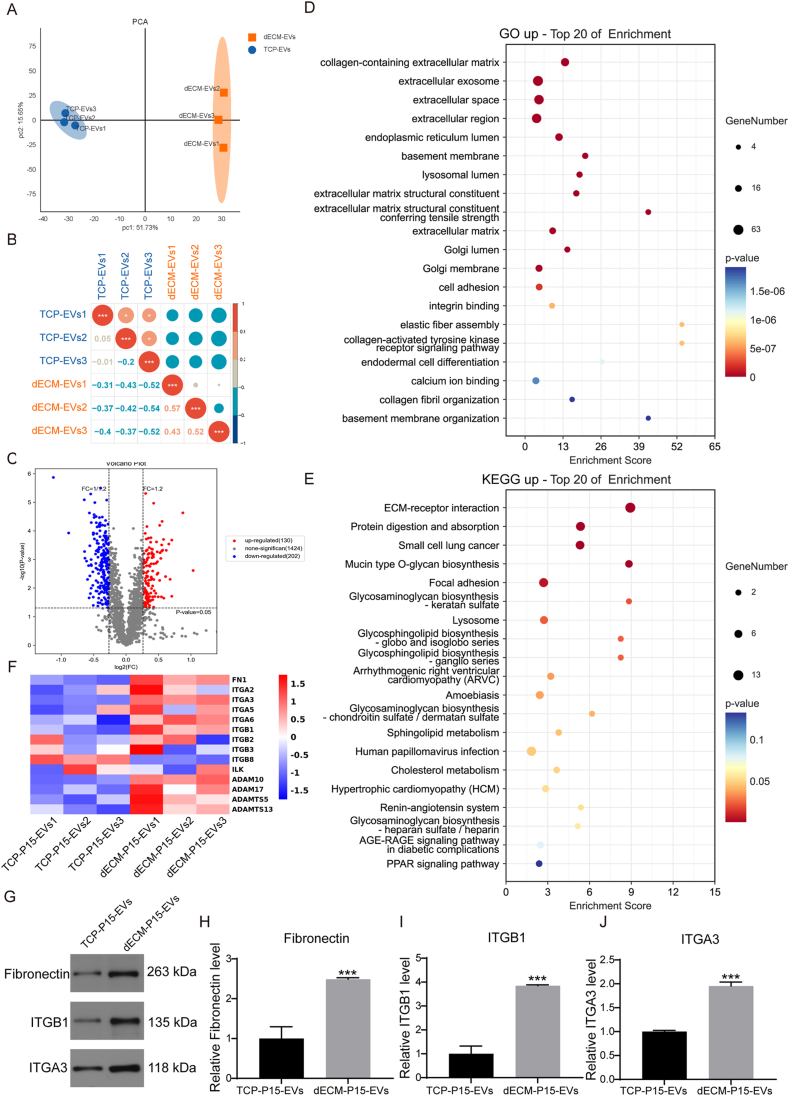
Proteomic profiling of TCP-P15-EVs and dECM-P15-EVs. (A) PCA analysis; (B) Sample correlation plot; (C) Volcano plot of differentially expressed proteins; (D) GO enrichment analysis of up-regulated proteins in dECM-P15-EVs; (F) KEGG enrichment analysis of up-regulated proteins in dECM-P15-EVs; (G) Western blot analysis of FN, ITGB1, and ITGA3 protein levels and (H–J) quantifications (n = 3). The statistical significance was assessed using two-tailed Student's unpaired t-tests. Data represent mean ± SD.

EVs from TCP- vs dECM-cultured hADSCs were lysed and digested with trypsin, and the resulting peptides were purified for liquid chromatography–mass spectrometry (LC-MS) and proteomic analysis. LC-MS quantified 2028 proteins (Supplementary Table 1). PCA and correlation plots confirmed variation in the protein abundances between triplicates of TCP-P15-EVs and dECM-P15-EVs (Fig. 4A and B; Supplementary Fig. 4A). DESeq2 identified 130 up- and 202 down-regulated proteins based on log2 fold-change≥1.2 or log2 fold-change≤1/1.2 and p-value<0.05 between TCP-P15-EVs and dECM-P15-EVs presented by Volcano plot and heat-map plot (Fig. 4C; Supplementary Fig. 4B). Functional enrichment analysis of up-regulated proteins in dECM-P15-EVs highlighted pathways including “integrin binding", “ECM structural constituent", “ER lumen", “collagen-containing ECM", “ECM-receptor interaction", “focal adhesion", and “glycosaminoglycan biosynthesis” (Fig. 4D and E). Down-regulated proteins in dECM-P15-EVs were enriched in “Ribosome” and “spliceosome pathways” (Supplementary Fig. 4CD), suggesting dECM-P15-EVs might alleviate chondrocyte senescence and ER-stress through delivering ECM proteins.

Consistent with the in vivo findings, proteomics analysis revealed up-regulation of FN, ITGA3, and ITGB1 in dECM-P15-EVs (Fig. 4F), which was confirmed by Western blot (Fig. 4G–J), supporting their possible role in mediating the anti-senescence effects of dECM-P15-EVs.

Additional ECM proteins—COL1A1, LAMA1, LAMA4, LAMA5, LAMB1—were up-regulated in dECM-P15-EVs (Supplementary Fig. 4E), and >50 % of their annotated GO terms aligned with enriched pathways (Fig. 4D), including “integrin binding”, “collagen-containing extracellular matrix”, etc. This result indicated that COL1A1 and LAMININ might synergistically participate in FN/ITGB1 axis regulation through Integrin binding and other pathways. “Laminin binding”, “collagen binding”, and “integrin-mediated signaling” were also increased (Supplementary Fig. 4F). STRING analysis positioned FN and ITGB1 at the network center (Supplementary Fig. 4G), underscoring the FN-ITGB1 axis in modulating EV function.

Collectively, dECM-P15-EVs may enhance chondrocyte resilience by enriching FN-integrin signaling cargo. we further investigated the key regulator of this cargo difference.

### 3.5 FN in dECM is pivotal for the anti-senescence effect of dECM-P15-EVs

**Fig. 5:**
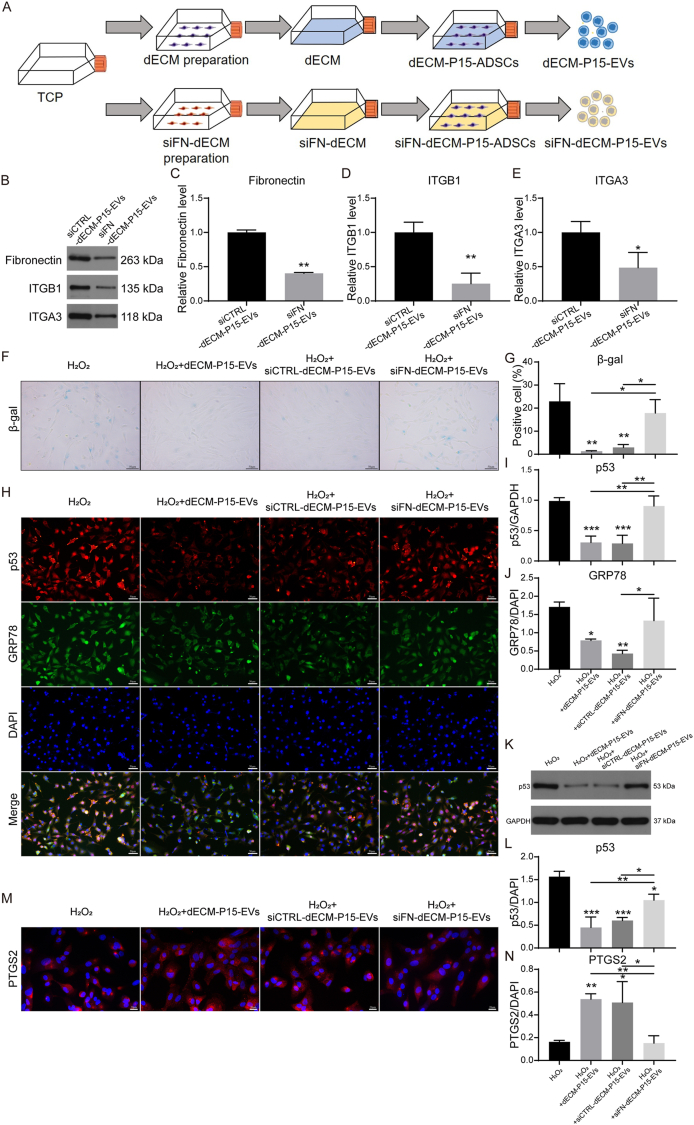
FN in dECM is pivotal for the anti-senescence effect of dECM-P15-EVs. Chondrocytes were treated with 100 mM H_2_O_2_ ± siCTRL-dECM-P15-EVs and siFN-dECM-P15-EVs. (A) Schematic of siFN-dECM-P15-EVs preparation; (B) Western blot analysis of FN, ITGB1, and ITGA3 protein levels in siCTRL-dECM-P15-EVs and siFN-dECM-P15-EVs and (C–E) quantifications (n = 3); Chondrocytes treated with 100 mM H_2_O_2_ ± 10 μg/ml dECM-P15-EVs, siCTRL-dECM-P15-EVs or siFN-dECM-P15-EVs.(F) β-gal staining of chondrocytes and (G) quantification (scale bar, 50 μm; n = 3); (H) Immunostaining of p53 and GRP78 in chondrocytes and (I, J) quantifications. DAPI labels nuclei (scale bar, 50 μm; n = 3); (K) Western blot of p53 protein level in chondrocytes and (L) quantification (n = 3); (M) Immunostaining of PTGS2 in chondrocytes and (N) quantifications. (DAPI labels nuclei; scale bar, 50 μm; n = 3). The statistical significance was assessed using one-way ANOVAs with Tukey's multiple comparison tests (∗*P* < 0.05, ∗∗*P* < 0.01, ∗∗∗*P* < 0.001). Data represent mean ± SD.

**Fig. 6:**
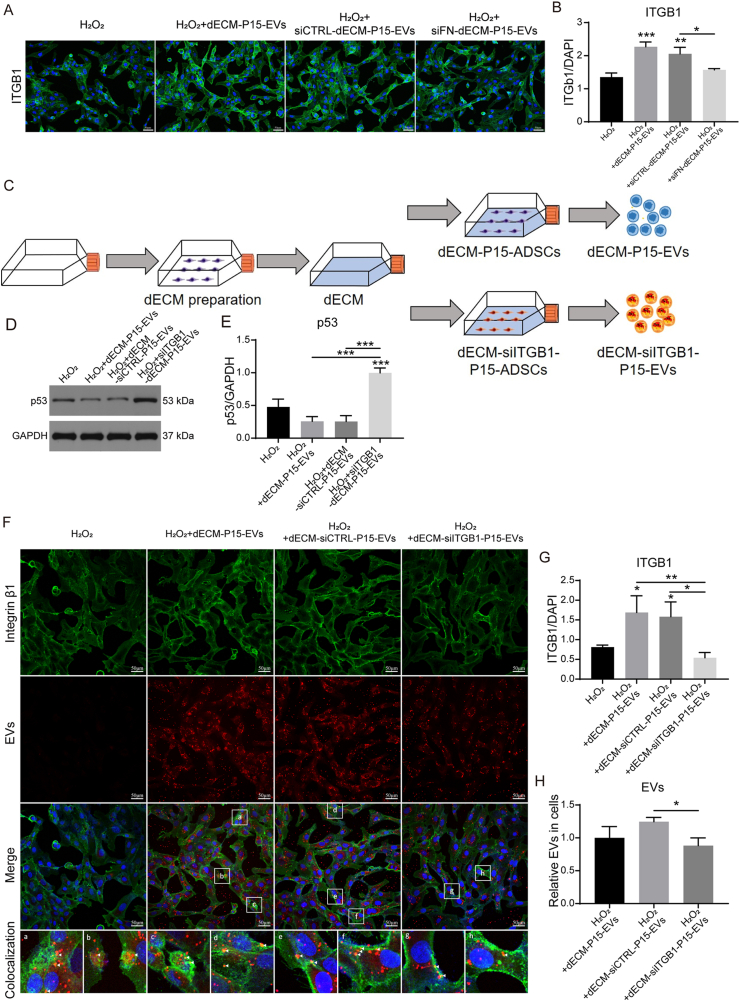
Exosomal ITGB1 is indispensable for the anti-senescence activity of dECM-P15-EVs. 100 mM H_2_O_2_-stimulated chondrocytes were treated with 10 μg/ml dECM-P15-EVs, siCTRL-dECM-P15-EVs or siFN-dECM-P15-EVs. (A) Immunostaining of ITGB1 in chondrocytes and (B) quantification (DAPI labels nuclei; scale bar, 50 μm; n = 3); (C) Schematic of dECM-siITGB1-EVs preparation; 100 mM H_2_O_2_-stimulated chondrocytes were treated with 10 μg/ml dECM-P15-EVs, dECM-siCTRL-EVs or dECM-siITGB1-EVs. (D) Western blot of p53 protein level in chondrocytes and (E) quantification. (GAPDH serves as loading control, n = 3); (F) Immunostaining of ITGB1 and EVs in chondrocytes and (G, H) quantifications of ITGB1 and intracellular EVs (DAPI labels nuclei, scale bar, 50 μm; n = 3). The statistical significance was assessed using two-tailed Student's unpaired t-tests or one-way ANOVAs with Tukey's multiple comparison tests (∗*P* < 0.05, ∗∗*P* < 0.01, ∗∗∗*P* < 0.001). Data represent mean ± SD.

Previous research demonstrated that expansion of high-passage MSCs on FN depleted dECMs resulted in decreased cellular proliferation, growth, and cell cycling. Recent study also demonstrated that elevated FN in ECM directly feeds back to enhance cellular FN synthesis and subsequent loading into EVs [39,40]. Therefore, we investigated whether FN depletion in dECMs impair the functional capacity of EVs derived from expanded hADSCs on them. Knockdown of FN in dECM-producing cells yielded siFN-dECM, on which hADSCs were cultured and EVs (siFN-dECM-P15-EVs) were isolated (Fig. 5A). TEM, NTA and Western blot showed no change in EV morphology or markers (Supplementary Fig. 5A–C). Yet uptake assays revealed markedly reduced EV accumulation within chondrocytes (Supplementary Fig. 5D–E). We therefore examined whether these EVs lack key cargo enriched in dECM-P15-EVs. Western blot confirmed that FN, ITGB1, and ITGA3 are markedly reduced in siFN-dECM-P15-EVs (Fig. 6B–E).

We next assessed whether siFN-dECM-P15-EVs retained the capacity to counteract senescence. β-gal staining and p53 quantification showed that, unlike dECM-P15-EVs and siCTRL-dECM-P15-EVs, siFN-dECM-P15-EVs failed to reduce senescence of chondrocytes (Fig. 5F–I, 5K-L) and instead elevated GRP78 (Fig. 5H and J). They also failed to restore the inflammation-modulating factor PTGS2 (Fig. 5M−N), underscoring an inability to curb senescence-associated inflammation. In summary, our findings demonstrated that the presence of FN in the dECM is indispensable for mediating the therapeutic superiority of EVs produced by expanded hADSCs.

### 3.6 Exosomal ITGB1 is indispensable for the anti-senescence activity of dECM-P15-EVs

Unlike dECM-P15-EVs or siCTRL-dECM-P15-EVs, siFN-dECM-P15-EVs, which were confirmed to carry less ITGB1, failed to restore ITGB1 expression (Fig. 6A and B), To further directly verify that exosomal ITGB1 is indispensable for the anti-senescence and anti-ER stress effect of dECM-P15-EVs, knockdown of ITGB1 in hADSCs maintained in dECM yielded dECM-siITGB1-ADSCs and EVs (dECM-siITGB1-EVs) were isolated (Fig. 6C). These vesicles neither reduced p53 activation nor exhibited any anti-senescence capacity compared with dECM-siCTRL-EVs (Fig. 6D and E). Confocal microscopy showed pronounced co-localization of Cy3-labeled EVs and FITC-ITGB1 (arrowheads) in chondrocytes treated with dECM-P15-EVs, whereas dECM-siITGB1-EVs failed to replenish ITGB1 expression and were poorly internalized (Fig. 6F–H). Collectively, these findings established exosomal ITGB1 as both cargo and conduit through which dECM-P15-EVs deliver their anti-senescence benefits.

### 3.7 FN/ITGB1 engagement is indispensable for the anti-senescence action of dECM-P15-EVs

**Fig. 7:**
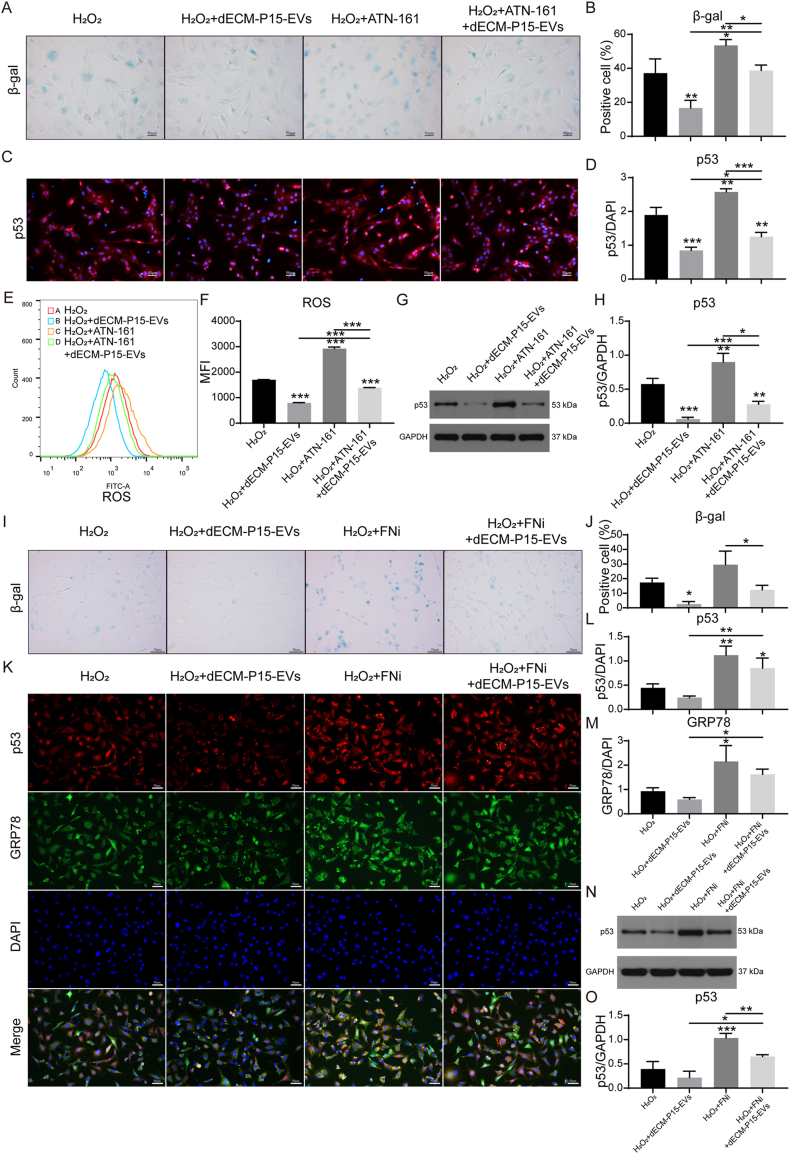
FN/ITGB1 engagement is indispensable for the anti-senescence action of dECM-P15-EVs. 100 mM H_2_O_2_-stimulated chondrocytes were treated with dECM-P15-EVs, ATN-161 (20 μM, 2 h), or dECM-P15-EVs + ATN-161. (A) β-gal staining of chondrocytes and (B) quantification (scale bar, 50 μm; n = 3); (C) Immunostaining of p53 in chondrocytes and (D) quantification (DAPI labels nuclei; scale bar, 50 μm; n = 3); (E) Flow cytometry of ROS level in chondrocytes and (F) quantification (n = 3); (G) Western blot of p53 and SIRT1 protein level in chondrocytes and (H) quantification. (GAPDH served as loading control; n = 3); 100 mM H_2_O_2_-stimulated chondrocytes were treated with dECM-EVs, FN inhibitor (FNi, sc-202156, 100 μM, 24 h), or dECM-P15-EVs + FNi. (I) β-gal staining of chondrocytes and (J) quantification (scale bar, 50 μm; n = 3); (K) Immunostaining of p53 and GRP78 in chondrocytes and (L, M) quantifications. (DAPI labels nuclei; scale bar, 50 μm; n = 3); (N) Western-blot of p53 protein level in chondrocytes and (O) quantification (GAPDH served as loading control; n = 3). The statistical significance was assessed using one-way ANOVAs with Tukey's multiple comparison tests (∗*P* < 0.05, ∗∗*P* < 0.01, ∗∗∗*P* < 0.001).

We next investigated the role of direct FN/ITGB1 engagement in the modulation of chondrocyte senescence. Pharmacological blockade of ITGB1 in recipient chondrocytes with the selective α5β1 antagonist ATN-161 partially abrogated the anti-senescence and ROS-scavenging capacities of dECM-P15-EVs (Fig. 7A–H). Complementarily, we disrupted FN fibrillogenesis and its subsequent integrin binding by treating cultures with the FN polymerization inhibitor sc-202156; this intervention completely abolished both the anti-senescence and ER-stress-alleviating effects of dECM-P15-EVs (Fig. 7I–O). Together, these findings establish that physical FN/ITGB1 engagement is a prerequisite for EV-mediated suppression of chondrocyte senescence.

### 3.8 SIRT1 functions as the essential downstream executor of ITGB1 in dECM-P15-EV-driven chondrocyte senescence suppression

**Fig. 8:**
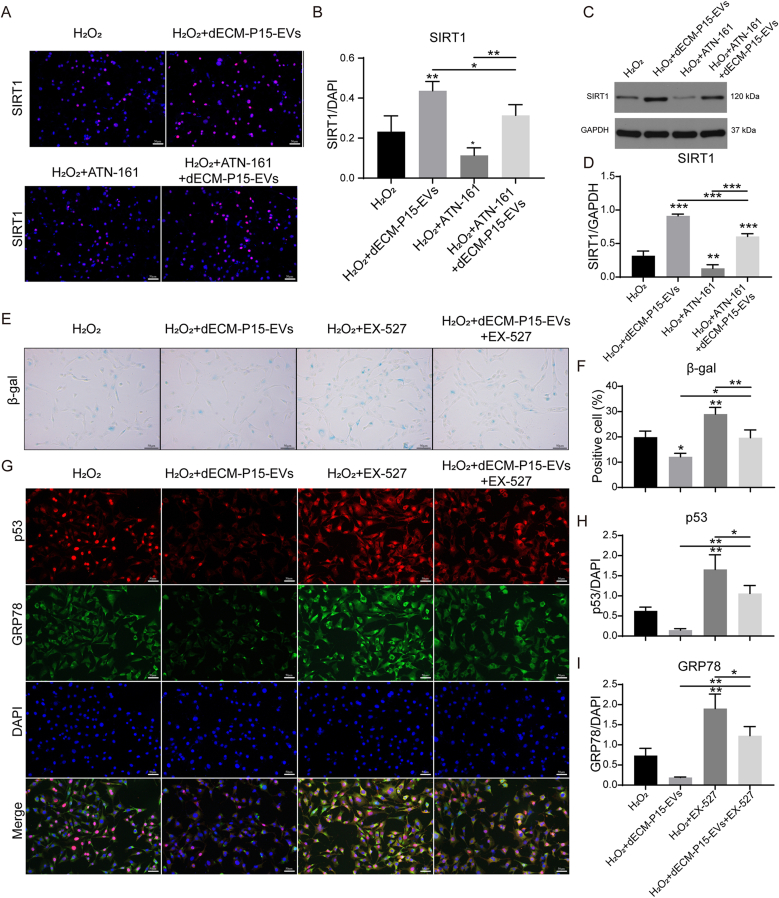
SIRT1 functions as the essential downstream executor of ITGB1 in dECM-P15-EV-driven chondrocyte senescence suppression 100 mM H_2_O_2_-stimulated chondrocytes were treated with dECM-P15-EVs, ATN-161 (20 μM, 2 h), or dECM-P15-EVs + ATN-161. (A) Immunostaining of SIRT1 in chondrocytes and (B) quantification. DAPI marks nuclei (scale bar, 50 μm; n = 4); (C) Western blot of SIRT1 protein level in chondrocytes and (D) quantification; (GAPDH served as loading control, n = 3); 100 mM H_2_O_2_-stimulated chondrocytes treated with dECM-P15-EVs, SIRT1 inhibitor EX-527(10 μM, 2 h), or dECM-P15-EVs + EX-527. (E) β-gal staining of chondrocytes and (F) quantification (scale bar, 50 μm; n = 3); (G) Immunostaining of p53 and GRP78 in chondrocytes and (H, I) quantifications. (DAPI marks nuclei; scale bar, 50 μm; n = 3). The statistical significance was assessed using one-way ANOVAs with Tukey's multiple comparison tests (∗*P* < 0.05, ∗∗*P* < 0.01, ∗∗∗*P* < 0.001).

To dissect the signaling cascade downstream of ITGB1, H_2_O_2_-stressed chondrocytes were pre-treated with the ITGB1 antagonist ATN-161, and SIRT1 expression was monitored by western blot and IF. Both assays revealed that dECM-P15-EVs robustly elevated SIRT1 levels; this increase was completely blocked by ATN-161 precondition (Fig. 8A–D). To interrogate the functional necessity of SIRT1, cells were co-incubated with dECM-P15-EVs and the selective SIRT1 inhibitor EX-527. EX-527 fully neutralized the anti-senescence and cytoprotective effects of the EVs (Fig. 8E–I), confirming that SIRT1 activation is indispensable for EV-mediated protection. Collectively, these data establish that SIRT1 serves as the downstream mediator of ITGB1 in dECM-P15-EV-driven inhibition of chondrocyte senescence.

## 4 Discussion

In this study, we showed that ER stress drove chondrocyte senescence, corroborating earlier reports of unresolved ER stress promoting senescence and apoptosis in osteoarthritic cartilage [41]. Pharmacological ER-stress inhibition restored ITGB1 expression, positioning ITGB1 as a critical regulator of ER stress, a finding consistent with its previously documented protective role against ER stress and apoptosis in high-glucose-stressed cardiomyocytes [36]. In articular cartilage, ITGB1 is indispensable for chondrocyte phenotype maintenance [42]. Human OA cartilage showed markedly reduced ITGB1 in severely lesioned areas [43], and conditional deletion of ITGB1 in Prx1-expressing cells decreased the non-calcified-to-calcified cartilage ratio while accelerating hypertrophy [42,44]. Recent work further demonstrated that ITGB1 on recipient cells is required for efficient EV uptake [45]. Our data align with these findings as suppressing ITGB1 in chondrocytes abolished the anti-senescence efficacy of dECM-P15-EVs, an effect mediated by the FN/ITGB1 axis. As the primary ligand for ITGB1, FN on EVs binds the leucine-aspartic acid-valine motif of α4β1 integrin; blocking this interaction markedly reduces EV internalization [46]. Consistently, dECM-P15-EVs are enriched in FN, and disrupting FN/ITGB1 engagement significantly impaired their anti-senescence effect. Thus, FN carried by dECM-P15-EVs and ITGB1 expressed on chondrocytes reciprocally interact to confer resistance against ER-stress-induced chondrocyte senescence.

hADSCs can protect against ER stress–related damage through both paracrine EVs and direct cell–cell contact [47,48]. Accumulating clinical evidence indicates that hADSC-based therapies are advancing toward clinical translation. Intra-articular injection of autologous and allogenic hADSCs provided both significant pain relief and functional improvements in OA patients [[49], [50], [51]]. EVs are potent intercellular mediators whose cargo mirror the functional state of their parental cells and dictate therapeutic outcome [52,53]. Strategies to enhance EV potency remain contentious: genetic engineering risks off-target alterations and cargo heterogeneity, whereas chemical surface modification can inactivate membrane proteins [54,55]. We therefore used dECM culture to rejuvenate EVs indirectly, optimizing the MSC niche without added biosafety concerns. dECM-P15-EVs markedly attenuated ER stress-induced senescence, whereas TCP-P15-EVs exacerbated it. This functional dichotomy aligns with observations that EVs isolated from osteoarthritic joints display impaired efficacy and exert deleterious effects on osteoclasts [56], underscoring the critical influence of the cellular microenvironment on EV composition and therapeutic performance. With comparable uptake efficiencies, the divergent anti-senescence activity of these two type of EVs must be cargo-driven. dECM-P15-EVs carried markedly higher FN and ITGB1 than TCP-P15-EVs. Integrins are well documented to be packaged into EVs, where they modulate cellular behavior [57], and EV-bound integrins facilitate uptake by engaging cognate ligands on target cells [58,59], thereby influencing biodistribution and tissue homing. We further observed that EVs can transfer ITGB1 to chondrocytes. Previous studies have demonstrated that EVs accomplish this “receptor-protein” intercellular transfer without the need for de novo transcription or translation, allowing immediate functional re-establishment on the recipient membrane. Tumor- and immune-cell-derived EVs, for instance, exhibit organotrophic trafficking dictated by their integrin repertoire [[58], [59], [60], [61]]. Consistent with these observations, we observed impaired chondrocyte uptake of ITGB1-knockdown EVs (Supplementary Fig. 6), and co-culture with these EVs resulted in diminished ITGB1 activation in chondrocytes, consistent with decreased ITGB1 delivery. This deficit also compromised the anti-senescence efficacy of dECM-P15-EVs, confirming that efficient intracellular ITGB1 transfer is required for their protective effect.

EV cargo is tightly dictated by the source cell; niche-derived signals profoundly influence cellular phenotype and fate, and consequently the composition of their EVs. In dECM-P15-EVs, the elevated ITGB1 content is linked to the abundance of FN within the dECM. Compared with TCP culture, the FN-abundant dECM microenvironment up-regulated ITGB1 expression of expanded cells, yielding a corresponding rise in ITGB1 within dECM-P15-EVs; knockdown of dECM-contained FN significantly reduced ITGB1 levels in dECM-EVs, impaired their ability to deliver ITGB1 to target cell, and ultimately abolished the anti-senescence therapeutic effect. Simultaneously, FN itself is more abundant in dECM-P15-EVs than in TCP-P15-EVs. A growing body of evidence demonstrates that elevated FN in dECM directly feeds back to enhance cellular FN synthesis and subsequent loading into EVs [39,40,62];. We therefore propose that FN in dECM interacts with ITGB1 on MSCs, driving FN transcription, translation, and secretion; newly synthesized FN is not only incorporated into the ECM but also packaged into EVs, thereby elevating both FN and ITGB1 in the resulting vesicles. Thus, EVs act not only as “couriers” for ITGB1 but also rely on the FN–ITGB1 axis to accomplish microenvironment-based cargo modulation and maximize therapeutic efficacy.

SIRT1, a NAD^+^-dependent histone deacetylase, counters aging by enhancing stress resistance and cellular metabolism [[63], [64], [65]]. Heterozygous SIRT1 mice spontaneously developed OA and other age-related defects, underscoring its indispensable role in physiological homeostasis [66]. Moreover, recent studies have identified SIRT1 as a key guardian against ER-stress-mediated organ damage [67], and have linked its expression to FN and integrin signaling [32,33,68]. Building on these findings, we showed that dECM-P15-EVs re-established FN/ITGB1 homeostasis and thereby up-regulated SIRT1; pharmacological blockade of SIRT1 abolished their capacity to mitigate ER stress and senescence, establishing the FN/ITGB1/SIRT1 axis as the core driver of their therapeutic effect.

## 5 Conclusion

In conclusion, this study shows that dECM-P15-EVs restore FN/ITGB1 homeostasis, reinvigorate SIRT1 signaling, and thereby ameliorate ER stress to block chondrocyte senescence and halt cartilage degradation. These findings position dECM-P15-EVs as a readily scalable, senescence-resistant biotherapy for translational OA treatment.

Limitation of the present study is the use of only adipose-derived MSCs and the relatively young (28–40 years) donors. We propose that dECM-based rejuvenation should be applicable to a broad range of MSC-EV based OA treatment. Future work should compare MSC-EVs from diverse sources, various donor age and disease status, and conduct comprehensive in vivo experiments to compare the functionality of these EVs with those derived from dECM-rejuvenated MSCs. Such investigations would help further solidify the conclusions of this study and fully illustrate the translational potential of dECM-based rejuvenation strategies.

## Declaration of generative AI in scientific writing

During the preparation of this work the authors used ChatGPT in order to assist in language polishing of the manuscript. After using this tool, the authors reviewed and edited the content as needed and take full responsibility for the content of the published article.

## Funding

This work was supported by grants from the 10.13039/100007219Shanghai Natural Science Foundation (23ZR1452400), the 10.13039/100017950Shanghai Municipal Health Commission Health Industry Clinical Research Project Youth Program (20244Y0060), the 10.13039/501100001809National Natural Science Foundation of China (82302766), the Cell and Gene therapy project of the Shanghai Science and Technology Innovation action plan (23J11900400), the Peak Disciplines (Type IV) of Institutions of Higher Learning in Shanghai, the Healthcare Talents Youth Program of Shanghai Pudong New Area (2025PDWSYCQN-01), the Shanghai East Hospital clinical research project (DFLC2022010), the Shanghai East Hospital characteristic professional subjects construction project (2024-DFTS-004).

## CRediT authorship contribution statement

Aoyuan Fan: Conceptualization, Funding acquisition, Investigation, Writing – original draft. Zhiying Pang: Formal analysis, Investigation, Visualization, Writing – original draft. Zheng Liu: Formal analysis, Validation, Writing – review & editing. Feng Yin: Conceptualization, Funding acquisition, Supervision, Writing – review & editing. Yiming Wang: Conceptualization, Funding acquisition, Supervision, Writing – review & editing.

## Declaration of competing interest

The authors declare that they have no known competing financial interests or personal relationships that could have appeared to influence the work reported in this paper.
