# Supplementary material for: Decellularized extracellular matrix restores Fibronectin/Integrin β1 balance through extracellular vesicles to rejuvenate chondrocytes and alleviate osteoarthritis progression
Source: J Orthop Translat. 2025 Dec 20;56:101019. doi: 10.1016/j.jot.2025.10.011 (PMC12988504; doi:10.1016/j.jot.2025.10.011)
Supplement: Multimedia component 1 [file mmc1.docx]

**Supplementary Figures**

Title: Decellularized extracellular matrix restores Fibronectin/Integrin β1 balance through extracellular vesicles to rejuvenate chondrocytes and alleviate osteoarthritis progression


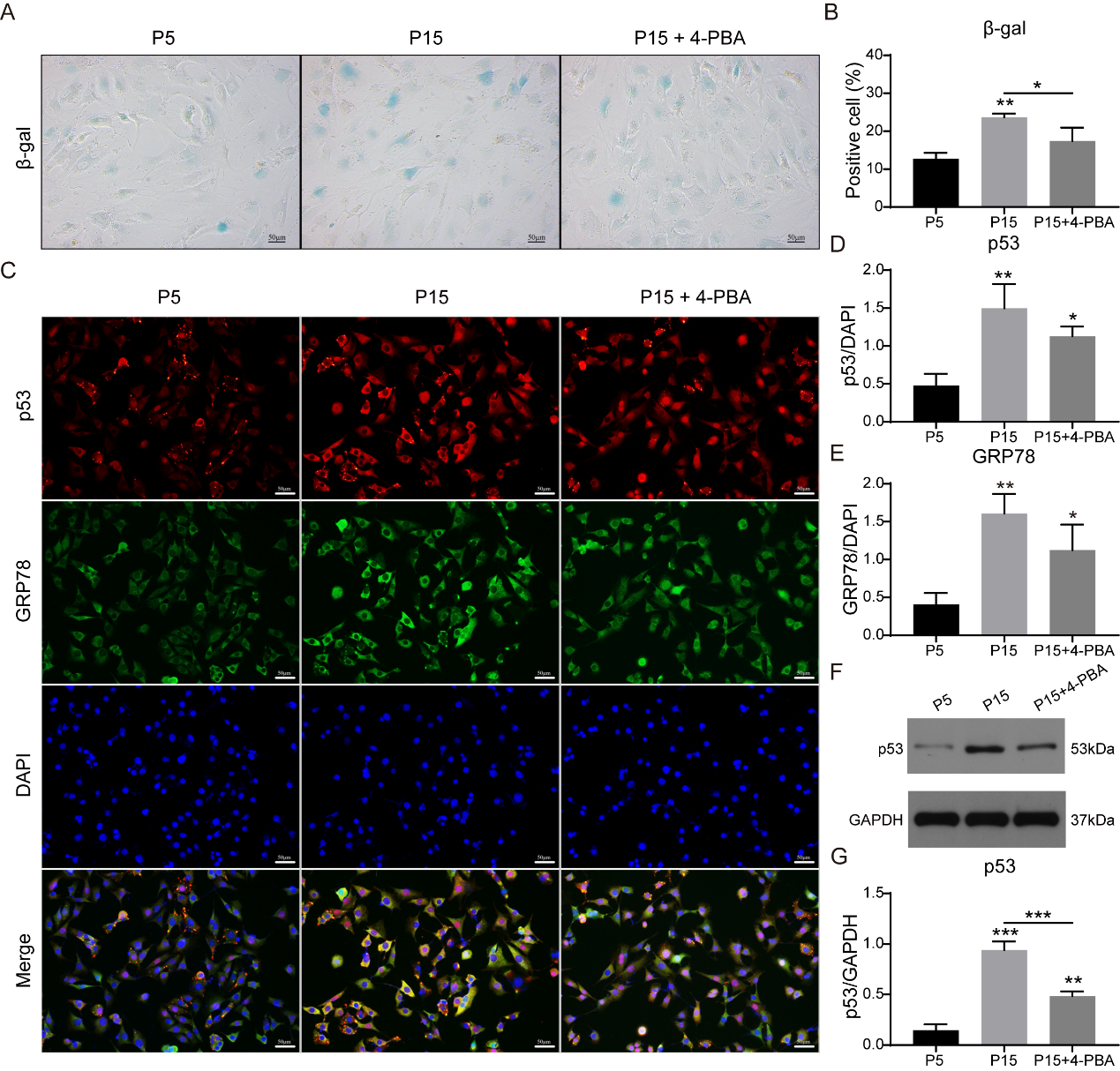


Supplementary Figure 1. ER stress inhibition mitigates passaging-induced chondrocyte senescence

Passage 5 (P5), passage 15 (P15) and P15 +200 ng/ml 4-PBA (200 ng/ml, 24 h) chondrocytes were assessed. (A) β-gal staining of chondrocytes and (B) quantification (scale bar, 50 μm; n=3); (C) Immunostaining of p53 and GRP78 in chondrocytes and (D, E) quantifications (DAPI labels nuclei; scale bar, 50 μm; n=3); (F) Western-blot of p53 in chondrocytes and (G) quantification (GAPDH serves as loading control; n=4). The statistical significance was assessed using one-way ANOVAs with Tukey’s multiple comparison tests (**P* <0.05, ** *P* < 0.01, *** *P* < 0.001).


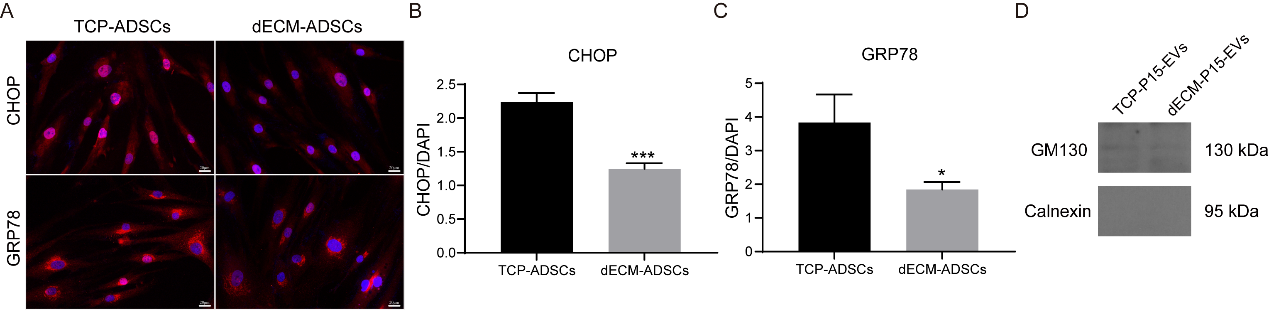


Supplementary Figure 2. dECM culture alleviates ER stress in hADSCs

P15 hADSCs cultured on TCP or dECM and TCP-P15-EVs and dECM-P15-EVs were isolated. (A) Immunostaining of CHOP and GRP78 in chondrocytes and (B, C) quantifications (DAPI marks nuclei; scale bar: 50 μm; n=3); (D) Western-blot of GM130 and Calnexin in chondrocytes. The statistical significance was assessed using two-tailed Student’s unpaired t-tests. Data represent mean ± SD (**P* <0.05, ** *P* < 0.01, *** *P* < 0.001).


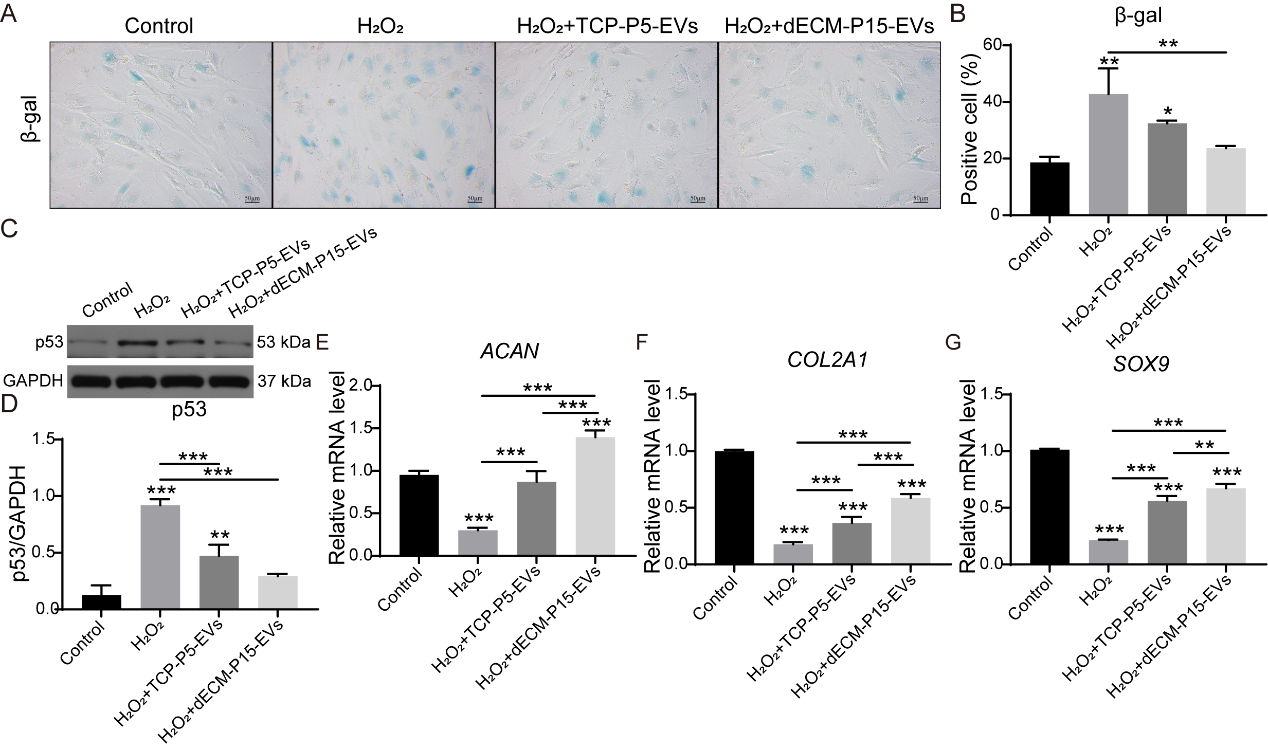


Supplementary Figure 3. dECM-P15-EVs and TCP-P5-EVs exert comparable anti-senescence effects

Chondrocytes were treated with 100 mM H₂O₂ ± 10 μg/ml TCP-P5-EVs or dECM-P15-EVs for 24 h. (A) β-gal staining of chondrocytes and (B) quantification (scale bar, 50 μm; n=3); (C) Western-blot analysis of p53 in chondrocytes and (D) quantification (GAPDH served as loading control; n=3); (E-G) qPCR of ACAN, COL2A1, and SOX9 mRNA levels in chondrocytes (n=3). The statistical significance was assessed using one-way ANOVAs with Tukey’s multiple comparison tests (**P* <0.05, ** *P* < 0.01, *** *P* < 0.001).

Supplementary Figure 4. Proteomic analysis of TCP-P15-EVs and dECM-P15-EVs

(A) Boxplot of both EVs; (B) Heatmap of differentially expressed proteins in both EVs;

(C) GO analysis of down-regulated pathway in dECM-P15-EVs; (D) KEGG analysis of down-regulated pathway in dECM-P15-EVs; (E) Heatmap of differentially expressed COL1A1 and LAMININ related proteins in both EVs; (F) GO analysis of ECM receptor related up-regulated pathway in dECM-P15-EVs; (G) String PPI analysis of ECM receptor related up-regulated proteins in dECM-P15-EVs.


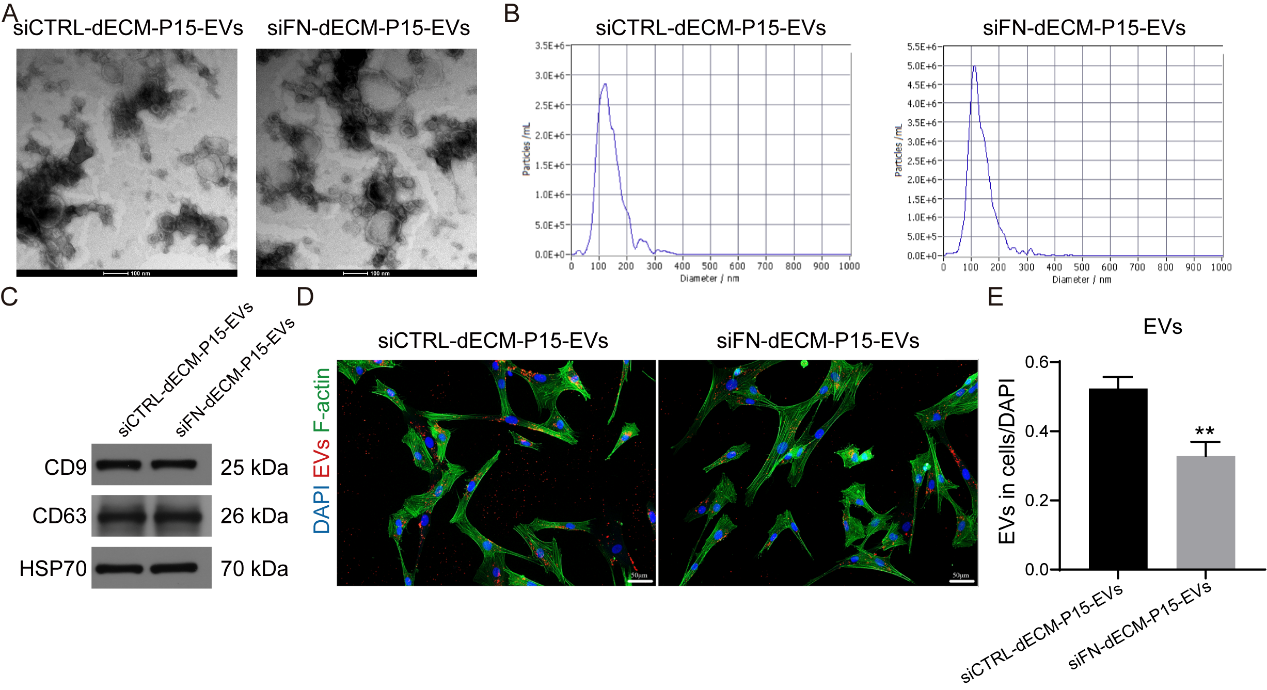


Supplementary Figure 5. FN knockdown in dECM impairs EV uptake

siCTRL-dECM-EVs and siFN-dECM-EVs were acquired. (A) TEM analysis of both EVs;

(B) NTA analysis of both EVs; (C) Western-blot of surface marker CD9, CD63, and HSP70 in both EVs; (D) Immunostaining of both EVs uptaken by chondrocytes and (E) quantification of intracellular EVs (DAPI labels nuclei; scale bar, 50 μm; n=3). The statistical significance was assessed using two-tailed Student’s unpaired t-tests. Data represent mean ± SD (**P* <0.05, ** *P* < 0.01, *** *P* < 0.001).


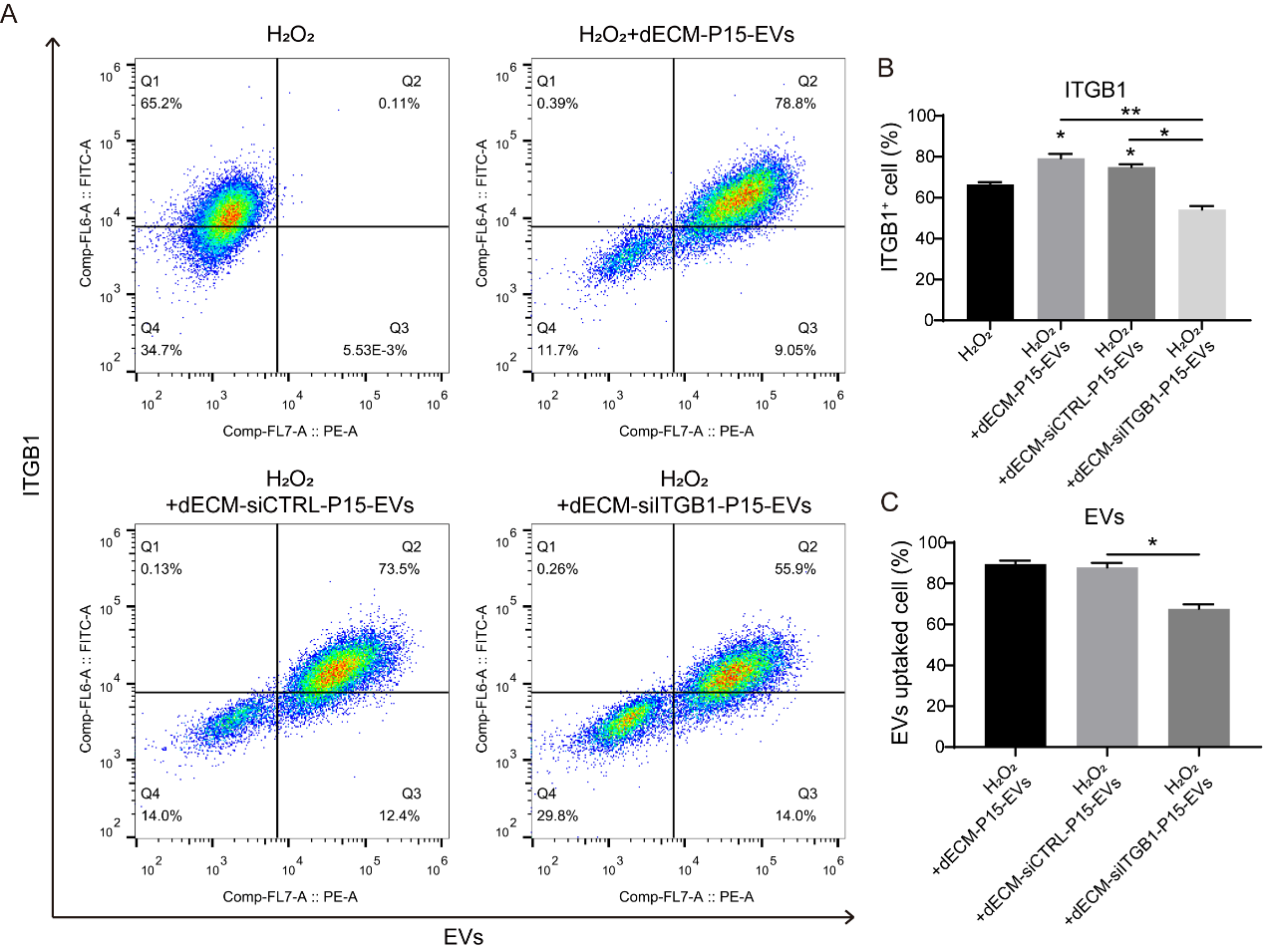


Supplementary Figure 6. FN inhibition impairs the therapeutic effect of dECM-P15-EVs

(A) Flow cytometry of chondrocyte treated with PKH26-labeled dECM-P15-EVs (n=3)

and (B, C) quantifications of ITGB1 positive cells and EVs positive cells. The statistical significance was assessed using one-way ANOVAs with Tukey’s multiple comparison tests (**P* <0.05, ** *P* < 0.01).
